# Limitations of haemozoin-based diagnosis of *Plasmodium falciparum* using dark-field microscopy

**DOI:** 10.1186/1475-2875-13-147

**Published:** 2014-04-17

**Authors:** Charles Delahunt, Matthew P Horning, Benjamin K Wilson, Joshua L Proctor, Michael C Hegg

**Affiliations:** 1 Intellectual Ventures Lab, Bellevue, WA, USA; 2 University of Washington, Seattle, WA, USA; 3 Institute for Disease Modeling, Bellevue, WA, USA

**Keywords:** Haemozoin, Hemozoin, Malaria pigment, Dark-field microscopy, Image-processing algorithms, Malaria diagnostics, Early ring parasites

## Abstract

**Background:**

The haemozoin crystal continues to be investigated extensively for its potential as a biomarker for malaria diagnostics. In order for haemozoin to be a valuable biomarker, it must be present in detectable quantities in the peripheral blood and distinguishable from false positives. Here, dark-field microscopy coupled with sophisticated image processing algorithms is used to characterize the abundance of detectable haemozoin within infected erythrocytes from field samples in order to determine the window of detection in peripheral blood.

**Methods:**

Thin smears from *Plasmodium falciparum*-infected and uninfected patients were imaged in both dark field (DF) unstained and bright field (BF) Giemsa-stained modes. The images were co-registered such that each parasite had thumbnails in both BF and DF modes, providing an accurate map between parasites and DF objects. This map was used to find the abundance of haemozoin as a function of parasite stage through careful parasite staging and correlation with DF objects. An automated image-processing and classification algorithm classified the bright spots in the DF images as either haemozoin or non-haemozoin objects.

**Results:**

The algorithm distinguishes haemozoin from non-haemozoin objects in DF images with an object-level sensitivity of 95% and specificity of 97%. Ring stages older than about 6 hours begin to show detectable haemozoin, and rings between 10–16 hours reliably contain detectable haemozoin. However, DF microscopy coupled with the image-processing algorithm detect no haemozoin in rings younger than six hours.

**Discussion:**

Although this method demonstrates the most sensitive detection of haemozoin in field samples reported to date, it does not detect haemozoin in ring-stage parasites younger than six hours. Thus, haemozoin is a poor biomarker for field samples primarily composed of young ring-stage parasites because the crystal is not present in detectable quantities by the methods described here. Based on these results, the implications for patient-level diagnosis and recommendations for future work are discussed.

## Background

Haemozoin is a chemically inert, insoluble crystalline dimer of ferriprotoporphyrin IX. The crystal is intrinsic to the malaria parasite and possesses many unique optical and magnetic properties. Many methods for detecting haemozoin have been proposed as an alternative to conventional malaria diagnostics. These methods include depolarization side scatter (DPSS) flow cytometry [1,2], Raman scattering [3-5], magneto-optics [6,7], magnetic aggregation [8], photoacoustics [9-11], optical spectroscopy [12], third harmonic generation [13,14], and dark-field microscopy [5,15-19]. A haemozoin-based diagnostic is desirable because it does not require the chemical staining or other sample preparation methods of manual Giemsa-stain microscopy that tend to lower the overall sensitivity and specificity of the test [20]. Haemozoin is also a potential target for *in vivo* diagnostic techniques because its unique linear and non-linear optical properties can be probed at wavelengths within the transparency window of human tissue.

Prior work has also highlighted some of the challenges facing haemozoin-based diagnostics, including an apparent lack of haemozoin in ring-stage parasites [1,17,21]. Furthermore, *Plasmodium falciparum* exhibits sequestration in the microvasculature [22-24] and, as a result, parasites found in peripheral red blood cells are typically young, ring-stage parasites that appear to lack detectable haemozoin. The amount of haemozoin in the peripheral blood is the sum of newly-produced crystals within infected erythrocytes and neutrophil-ingested crystals. The spleen and liver (macrophages) remove haemozoin from ruptured schizonts effectively so there is little accumulation in the peripheral blood, aside from infected RBCs (iRBCs). Despite this knowledge, haemozoin is experiencing a renaissance within the malaria diagnostics community. Remarkably, the pace of haemozoin research is in fact accelerating, with at least 10 papers on haemozoin diagnostic techniques published in the last two years [1-3,7,9-11,14,21,25]. In light of this resurgence, it is timely to reconsider haemozoin as a biomarker for malaria and its suitability as a basis for diagnosis.

It is important to note that the notion of detectable amounts of haemozoin is tightly bound to the detection method. The available evidence suggests either that young ring-stage parasites lack crystalline haemozoin, or that rings lack sufficient haemozoin for the particular detection methods employed. For example, Rebelo *et al.* suggest that haemozoin begins to be detectable at 18 hours using flow cytometry, but not before [1]. It has been difficult to associate detectable haemozoin with well-defined ring stages because the *Plasmodium* populations used in studies are often not synchronized enough to achieve the time resolution required. Thus, the question of when rings first contain haemozoin crystals remains open. As evident in the recent literature, researchers are either unaware of the intermittent nature of haemozoin in the peripheral blood, or are operating under the implicit hypothesis that haemozoin exists in early ring-stage parasites, but in amounts that require a more sensitive method to detect.

Here, the value of haemozoin as a biomarker for diagnosing malaria using dark-field microscopy is assessed, which is arguably the gold standard for haemozoin detection. Haemozoin shows up particularly well in dark-field (DF), in which the target is illuminated in such a way that only scattered light is captured by the collector. DF exploits the distinctive light-scattering properties of haemozoin relative to the weak scattering properties of haemoglobin [19]. Malaria diagnostics targeting haemozoin with DF microscopy were first investigated in the 1930s [15], and again in the 1980s [16-18]. Relatively complicated and costly at the time, DF microscopy never achieved practical implementation as a malaria diagnostic. However, the recent advances in powerful, low-cost opto-electronics and software have ushered in a new era of microscopy-based diagnostics [26] and allowed revisiting DF microscopy as a potential malaria diagnostic. It has been previously shown that haemozoin crystals exhibit strong backscattering under DF reflection mode microscopy when coupled with cross-polarization [19], similar to DPSS flow cytometry but with higher SNR. This method has inherently high specificity due to the unique optical collection system, but still retains some false positives. However, there is no equivalent to Giemsa that is specific for haemozoin and it is difficult, if not impossible, for a microscopist to distinguish haemozoin from false positives during manual microscopy. Thus, any practical implementation of a dark-field haemozoin detector must include an image-processing algorithm to classify haemozoin and non-haemozoin objects.

In order for haemozoin to be of value as a biomarker, it must be distinguishable from false positive (FP) objects using automated classification algorithms, analogous to the staining techniques used to image parasites directly, and it must be present in detectable quantities in circulating erythrocytes. This paper describes experiments detecting haemozoin using a DF system and image-processing algorithms that distinguish haemozoin from distractor objects. The experimental protocol produced matched unstained DF and Giemsa-stained bright-field (BF) images, allowing direct comparison of stained parasites and DF objects. This enabled a thorough characterization of the abundance of haemozoin in infected erythrocytes as a function of parasite age. This paper reports two results: That haemozoin can be distinguished from false positives in dark-field with high sensitivity and specificity; and that haemozoin is not present in detectable quantities in early ring-stage parasites.

## Methods

The experimental setup was designed to assemble a collection of paired BF and DF images for various objects, including parasites (in BF), haemozoin objects (in DF), and non-haemozoin objects (in DF). This collection of paired images allowed staging of parasites via BF images, and provided a well-labeled set of DF objects for development of an automated haemozoin detection algorithm.

Blood smears from febrile patients were provided by Shoklo Malaria Research Unit in Thailand. The samples had been tested for malaria using three methods: rapid diagnostic tests (SD Bioline Malaria Ag P.f/Pan), Giemsa-stained microscopy, and polymerase chain reaction (PCR). The present experiment used only *P. falciparum*-positive samples, with no co-infection, and *Plasmodium*-negative samples (as confirmed by all three diagnostic methods). A total of 32,000 red blood cells (RBCs) from 23 samples (10 positive, 13 negative) were imaged. From this RBC sample set, 974 objects were found in DF and 160 parasites were found in BF after staining. The positive samples had a relatively high parasitaemia (as determined by microscopy), ranging from 43,000 to 290,000 parasites/μl, or about 8.6 to 58 parasites per 1,000 RBCs (assuming 5 × 10^6^).

Unstained thin smears, fixed in methanol, were first imaged using DF microscopy, then Giemsa-stained and imaged using BF microscopy. These images were then aligned with software and used to determine whether a given object observed under DF corresponded to a parasite and, conversely, whether a given parasite generated a detectable DF object. Imaging Giemsa-stained smears directly with DF microscopy is unworkable, as the Giemsa stain itself scatters light efficiently and thus shows up under DF [18].

All images were obtained using a Malvern Morphologi G2 microscope with a Baumer FWx20c colour camera (Baumer, Ltd., Southington, CT, USA). For each positive sample, about 1,000 RBCs were imaged. This is a sufficient number of cells to accurately reflect the profile of the parasite load, given the high parasitaemia of these samples and assuming a uniform distribution of parasites throughout the blood sample.

Unstained smears were fixed in methanol and then coated in a very thin layer of microscope immersion oil and imaged without coverslip under epi-illumination DF using a 50× Nikon 0.55 NA objective (Nikon Instruments, Inc., Melville, NY, USA). Nine overlapping 50× images were acquired. Immediately after DF imaging, the oil was removed by re-immersing in methanol and the smears were stained in a 10% solution of Giemsa (Sigma-Aldrich Corporation, St. Louis, MO, USA) in deionized water for 10 minutes. Forty-two overlapping BF images were obtained using an oil-immersion 100× Nikon 1.25 NA objective (Nikon Instruments, Inc., Melville, NY, USA), covering the DF 50× fields of view. For each negative sample, sixteen 50× DF images consisting of approximately 1,700 total blood cells were acquired. The CCD camera must be set to avoid pixel saturation in DF images, because saturation adversely affects color detection by the automated algorithm (see Additional file 1 for details).

The unstained DF images and stained BF images were registered using alignment algorithms. Briefly, a Hough transform circle detection algorithm was used to find the centers of the RBCs in every image (both BF and DF). Correlation filter methods were then used on the (appropriately scaled) RBC center locations to calculate the offsets between the various images.

**Figure 1:**
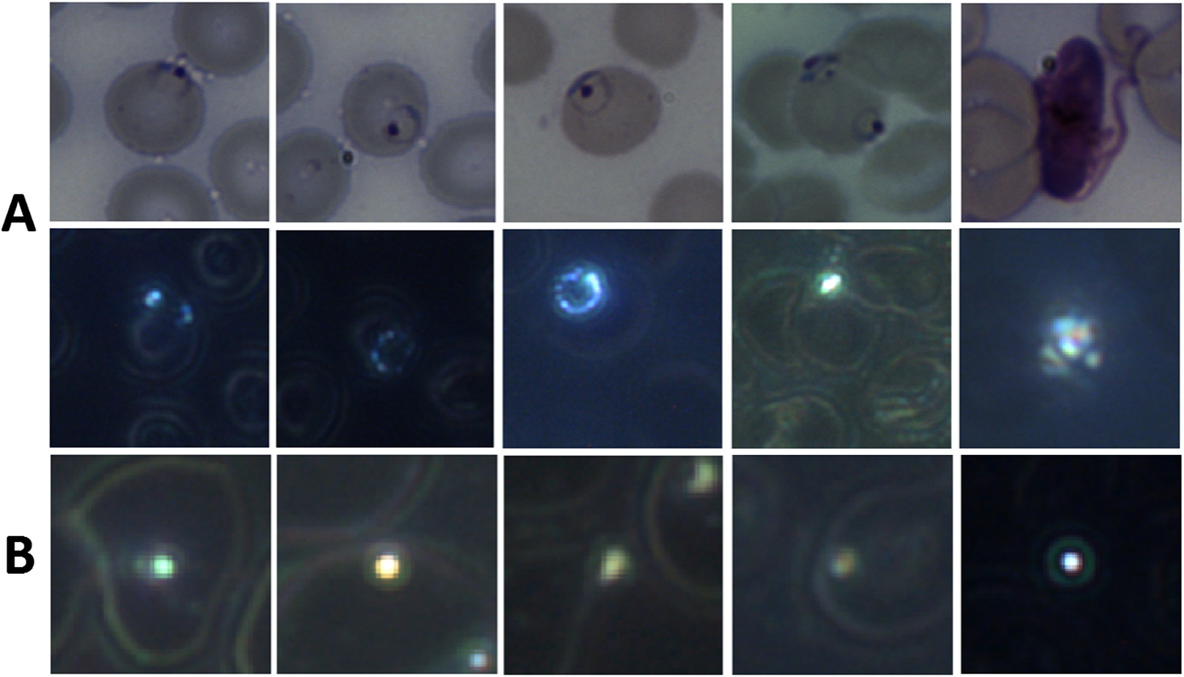
**Typical DF haemozoin and non-haemozoin objects.** Typical objects of interest. **A**: Haemozoin objects, 100× BF thumbnails (top row) and the corresponding 50× DF thumbnails (second row) showing the haemozoin within the parasites. (1–4) are rings, (5) is a gametocyte. Note in (4) that one ring/troph has visible haemozoin, while the other ring does not. **B**: Typical non-haemozoin objects, DF thumbnails. The larger rings are cell wall information due to haemoglobin scattering.

**Figure 2:**
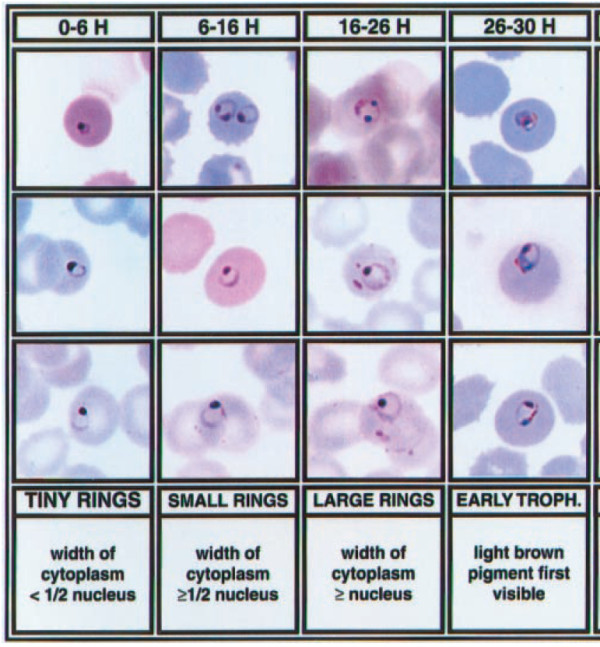
**Template for staging rings.** The template for staging rings into 0–6 hours, 6–16 hours, and 16–26 hours. From [21].

Every object of interest (e.g. a bright spot in DF or a parasite in BF) was thus represented by matching thumbnail images in both BF and DF modalities. In particular, each bright spot detected in the 50× DF images was linked to a stained BF 100× thumbnail (see for example Figure 1), while each parasite detected in the stained image was linked to a 50× DF thumbnail. This pairing of DF and BF thumbnails allowed construction of an accurate truth table: Each DF bright spot was labeled as positive (associated with a parasite in BF) or negative (no parasite); and each parasite was labeled as either associated with a DF bright spot or not. Giemsa-stain reading was performed by eye on the 100× BF images and parasites were staged according to Silamut *et al.*[22], using the template shown in Figure 2.

### Dataset statistics

A fully automated image-processing algorithm was applied to a labelled dataset of DF objects. This dataset consisted of 974 thumbnail DF images of objects of interest, drawn from DF 50× images obtained as described above. The objects were “bright spots”, selected by applying a threshold filter to a grayscale image of each sample. Due to variations in the brightness of the cell membrane and background, the threshold for each sample was adaptively chosen to be higher than the brightness of cell information due to haemoglobin scattering. The adaptive threshold ensured that most bright spots (i.e. spots with a signal stronger than the cell wall signal) were captured, while limiting the number of negative objects.

The dataset contains 51 haemozoin objects and 923 non-haemozoin objects (i.e. objects with no corresponding parasite in its BF image). Figure 1A and B show examples of both haemozoin and non-haemozoin objects imaged in DF. The non-haemozoin objects include bubbles, dust particles, and other unidentified artifacts. The haemozoin objects correspond to 41 mid-late rings (*>*6 hours), and 10 trophozoites and schizonts. Although some of the blood samples contain many early rings (0–6 hrs), these lack sufficient haemozoin to be detected (see Results) and thus did not have corresponding haemozoin objects.

The BF 100× images contained 160 parasites, including 150 rings. Of these rings, 41 had an associated DF 50× bright spot as described above. 109 rings had no associated DF 50× bright spot, and were thus invisible to the DF detection system.

### Algorithm outline

The purpose of the experiment was to assess the value of haemozoin as a biomarker in two ways: first, by determining if haemozoin can be distinguished from false positives by an automated image-processing algorithm; and second, by characterizing the abundance of haemozoin in early ring-stage parasites. The goal of the image-processing algorithm was to classify bright spots seen in DF as haemozoin or non-haemozoin. The algorithm is fully described in Additional file 1 and only briefly described here. The algorithm was developed using supervised learning on the fully labelled training set. It exploits both the shape and colour of the haemozoin signal in DF. The time required to image 1,000 cells and process the data with the complete algorithm is ~10 s, although this has not yet been optimized for speed.

### Shape

Haemozoin is deposited irregularly in the parasite, and because non-haemozoin objects are typically radially symmetric (i.e. close to circular), one branch of the algorithm measures how radially symmetric an object is. Haemozoin is typically asymmetric.

### Colour

Haemozoin scatters predominantly blue light due to its size and dielectric properties [19], while non-haemozoin objects scatter more broad-band light. The second part of the algorithm maps object colour into HSV space (Hue-Saturation-Intensity, an alternative colour basis to RGB). Haemozoin objects cluster closely together in the saturated blue region of HSV colour space.

This algorithm ignores gametocytes, for two reasons. First, gametocytes’ haemozoin signature is not amenable to detection by the algorithm’s particular colour analysis (see Additional file 1 for details). Second, gametocytes are very distinctive in DF (see Figure 1, row 2, column 5) and are thus easily detectable by other means.

## Results

This section first describes the object-level classification results of the algorithm. It then describes findings concerning when haemozoin (detectable by DF) first appears in ring forms.

### Effectiveness of DF haemozoin detection

**Figure 3:**
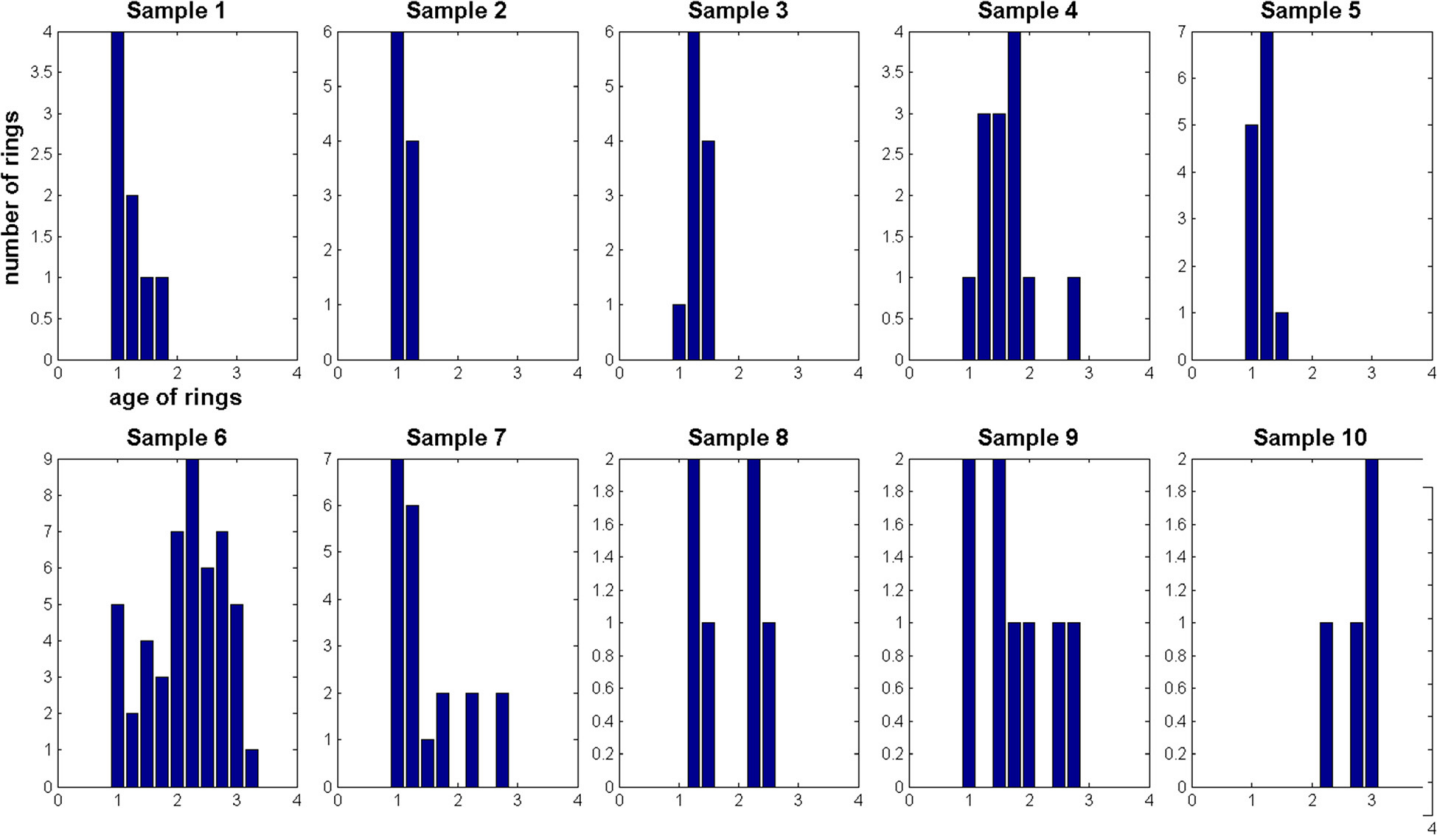
**Age distributions of rings by sample.** Age distribution of ring stages by sample. For each sample, the x-axis is the age of rings as follows: 1 = 0–6 hours, 2 = 6–16 hours, 3 = 16–26 hours; the y-axis shows the number of rings. Samples 1–5 and sample 10 show high synchronization. Samples 1–5 contained no detectable haemozoin objects.

The dataset of 974 DF objects was randomly divided into training (70%) and test (30%) sets (80 iterations) [27]. After determining the parameters of the algorithm with the training set, the algorithm was then used to classify the test set. On the test set the algorithm detected haemozoin with mean sensitivity 94.5% (std dev = 7.2%) and mean specificity 97.4% (std dev = 1.0%). Thus the DF method and algorithm identified haemozoin accurately. No false positives were observed in the negative samples; thus, the current noise floor is zero. Note, however, that this is somewhat artificial due to the low number of samples tested and one should expect a non-zero false positive rate upon further testing. Most importantly, however, more than half the ring parasites contained no detectable haemozoin signal and were thus invisible to this DF method. Of the 10 infected samples imaged, five contained only early rings with no haemozoin signal. Figure 3 shows the distribution of parasite age by sample. Samples 1–5 show a high degree of synchronization, with almost all parasites between 0–10 hrs old and none of these parasites containing detectable haemozoin. The implications of these results are discussed later.

### Absence of haemozoin in early rings

The classification algorithm described here assumes the parasite contains enough haemozoin to give a signal stronger than that given by the cell walls. That is, it assumes the haemozoin signal can be detected by the initial intensity thresholding. The sample images contain a total of 150 ring forms, as found in the 100× BF images using a combination of BF parasite detection algorithms and manual confirmation. However, most of these are early rings that lack sufficiently strong haemozoin signal and are thus invisible to the algorithm.

**Figure 4:**
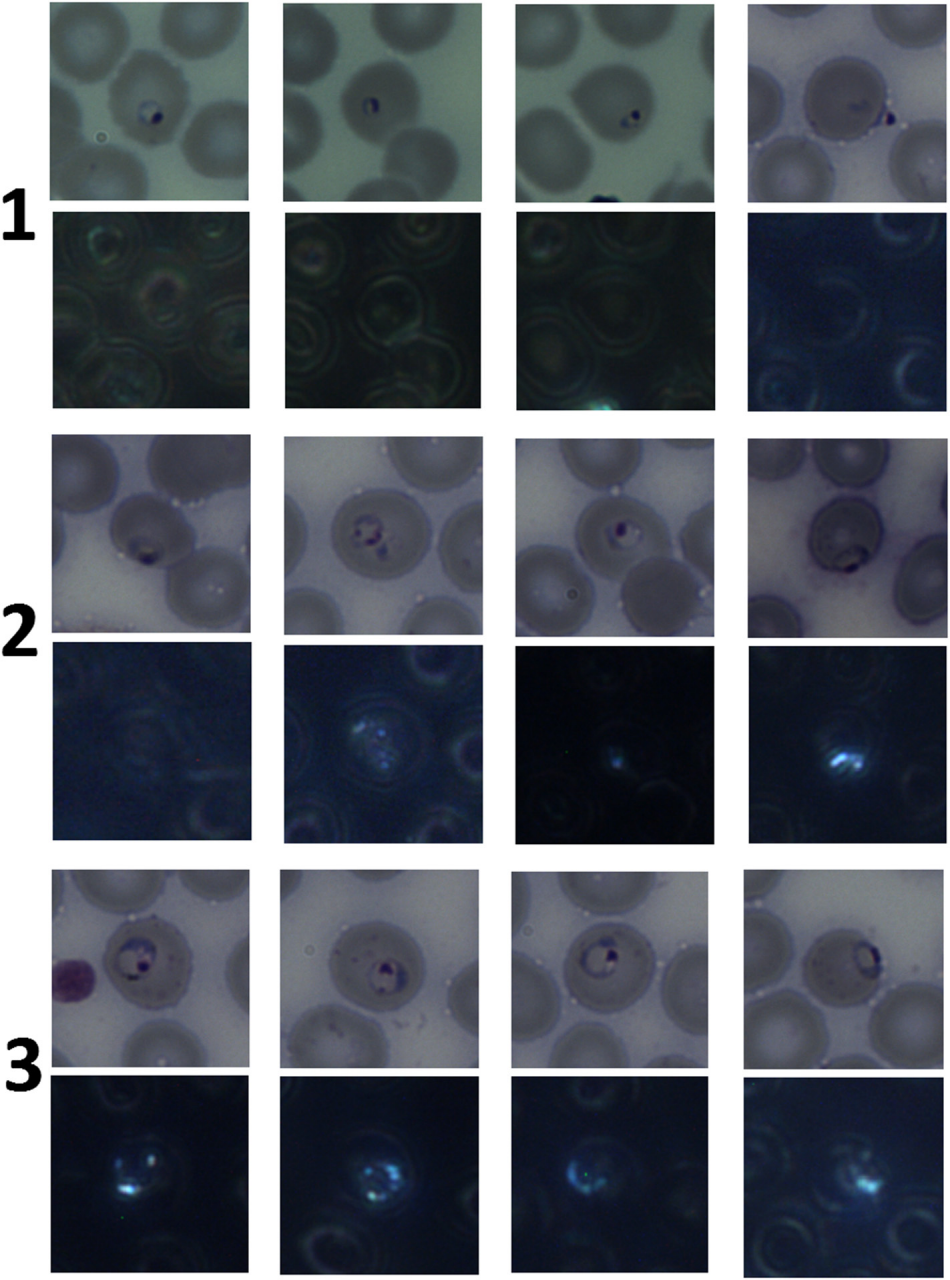
**Examples of ring stage parasites, BF and DF images.** Typical examples of ring stages, BF and corresponding DF. Row 1 = 0–6 hours old, row 2 = 6–16 hours old, row 3 = 16–26 hours old. Haemozoin is visible in none of row 1, most of row 2, and all of row 3.

To quantify the timing of when haemozoin first appears, all ring forms in the samples were staged by four of the authors (CD, MCH, MPH, BW). Figure 2 shows the template and criteria used for staging, from [22], which divides ring (i.e. immature trophozoite) stages into three windows: 0–6 hours, 6–16 hours, and 16–26 hours. Although staging parasites by eye is intrinsically subjective, a nominally finer age resolution can be achieved by averaging the four observers’ stagings, allowing indications of “toward the younger end” or “toward the older end” of a given window. Figure 4 shows some examples of rings of varying ages: In row 1, there is no visible haemozoin associated with the parasites; row 2 shows some visible haemozoin in slightly older rings; row 3 shows mature rings with easily detectable haemozoin.

**Figure 5:**
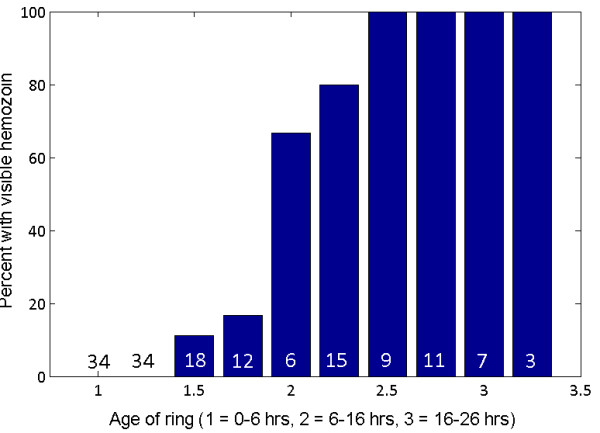
**Percentages of parasites with haemozoin *vs *age.** The percentage of various ages of ring parasites containing sufficient haemozoin signal to be detectable by the adaptive threshold test. The figure in the base of each column is the number of rings of that age in the sample set. On the age (*x-*) axis, 1 = 0–6 hrs, 2 = 6–16 hrs, 3 = 16–26 hrs. Values between these three integers correspond to split decisions among the observers and reflect edge cases. For example, 1.5 corresponds to the earlier end of 6–16 hrs, while 2.5 corresponds to the later end of 6–16 hrs.

Figure 5 shows the three main behaviors of ring-stage parasites in terms of haemozoin:

1. No early rings (*<*6 hrs) contain detectable levels of haemozoin.

2. About 70% of the mid rings (6–16 hrs) have detectable levels of haemozoin, with the percentage increasing with age through this range.

3. 100% of rings at the older end of 6–16 hrs and *>*16 hrs have detectable haemozoin. This suggests that under DF imaging, after roughly 6–10 hours haemozoin first begins showing up in large enough quantities to give a detectable signal stronger than the RBC cell walls, and after roughly 12 hours haemozoin is consistently giving a distinctive signal.

In addition, other colour-based algorithms were developed that detect possible (much weaker) haemozoin signal in DF images of about 20% of those rings that do not give bright spots. While this may indicate that small amounts of haemozoin are present prior to being detectable by the algorithm reported in this paper, it is unclear whether the detected signal was haemozoin or cell wall information caused by parasite-induced deformation of the RBC. These algorithms and results are not described here.

## Discussion and conclusions

The original purpose of this work was to determine the value of haemozoin as a biomarker for malaria through careful characterization of the abundance of detectable haemozoin in iRBCs and development of an automated pattern classification algorithm to distinguish haemozoin from false positives. When sufficient haemozoin was present to produce a signal above the RBC background signal, the algorithm presented here demonstrated excellent object-level sensitivity and specificity on 974 objects from 23 samples (10 positive, 13 negative). Although a relatively high number of non-haemozoin objects produced a detectable signal (923 objects), the algorithm rejected the vast majority of these, including all objects detected in negative samples. All false positives (objects classified as haemozoin but not associated with a parasite) were from three positive samples, implying that those objects may have actually been haemozoin, e.g. from ruptured cells. Furthermore, because of the lack of FPs in negative samples, the noise floor cannot adequately be characterized and thus it must be concluded for the moment that the algorithm is highly specific for the limited number of RBCs imaged per sample. This high specificity is a direct result of the physical characteristics of haemozoin which allows for easy classification; nevertheless, one would expect a non-zero noise floor as more RBCs are imaged. Note that the algorithm’s high sensitivity and specificity refer to detection of haemozoin objects, not to diagnosis of patients or even to detection of parasites (due to lack of haemozoin in early rings). Because the presentation of a given parasite is essentially the same whether from a high- or low-parasitaemia sample, this object-level sensitivity does not depend on the fact that all of the positive samples were high parasitaemia. However, the lack of haemozoin in early rings has implications for the sensitivity of patient- level diagnosis in low-parasitaemia samples, as discussed below.

The results demonstrated here represent the earliest detection of haemozoin in iRBCs to date. As mentioned previously, Rebelo *et al.* report noticeable amounts of haemozoin after 18 hours, but not at 12 hours [1]. The results indicate substantial haemozoin at 12 hours, which is consistent with [17], and haemozoin is often detected as early as six hours. Nevertheless, this detection method is limited by the apparent lack of detectable haemozoin in rings younger than six hours.

**Figure 6:**
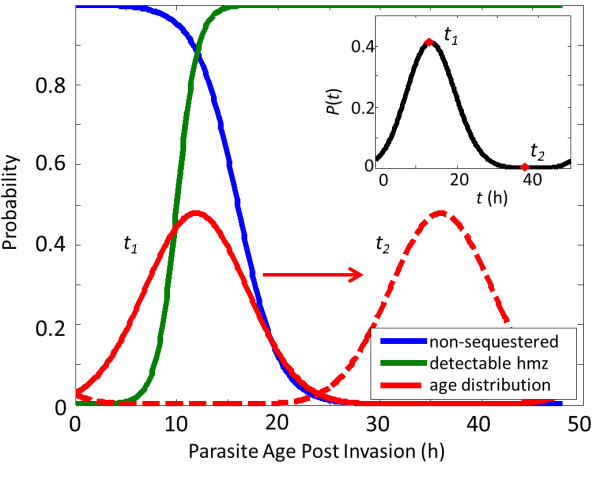
**Haemozoin peripheral blood dynamics.** The probability densities for haemozoin (green), sequestered parasites (blue), and parasite age distribution (red) as a function of time post-invasion. The inset shows the probability of detecting an individual parasite (black) vs. time during the cyclical infection when blood is drawn.

The main implications for these results can be understood in terms of the haemozoin dynamics in the peripheral blood. Figure 6 demonstrates the presence of a temporal haemozoin detection window bounded by a lack of haemozoin at young parasite ages and sequestration at older parasite ages. The blue curve shows the probability of a parasite not being sequestered as a function of parasite age [23], while the green curve shows the probability of a parasite containing detectable amounts of haemozoin, representing the results from the previous section. The red curve shows a hypothetical distribution of parasite ages within an infection. This can be represented with a moderately wide gaussian because it captures all of the relevant haemozoin dynamics, but the distribution could be multi-modal, non-gaussian or, in the case of highly synchronized infections, very narrow. Multiplying the blue and green curves together yields the distribution of available (in the peripheral blood) and detectable (containing haemozoin) parasites as a function of parasite age. When this new distribution is convolved with the red curve (parasite age distribution), the result is the black curve in the inset of Figure 6. This represents the probability that an individual parasite from this age distribution will be detected as a function of time. Note that the time axis of the inset is not parasite age (as in the main plot), but instead represents the time during the cyclical infection when a blood sample is taken. Thus, the model shows the detection window and probability of detection for a given parasite age distribution, subject to the constraints of lack of haemozoin and sequestration.

The probability of detection varies greatly and significantly depends on when peripheral blood is drawn from the patient. Furthermore, the probability of detection is less than 5% for over half of the 48-hr intraerythrocytic life-cycle, implying the need to image 20 times as many iRBCs as would be necessary to achieve the same detection limit with Giemsa. As previously discussed, the lack of detectable haemozoin in early rings is especially important for *P. falciparum*, where a high degree of synchronization (i.e. a much narrower age distribution) makes it likely that a prohibitive fraction of presenting infections will contain only early rings. For example, in the field samples that were imaged, all of which had high parasitaemia and might thus be expected to have less tightly synchronized parasite populations, five out of 10 contained no detectable haemozoin. It is possible that by observing more RBCs one could detect parasites in all positive samples. Even in this case, however, diagnosing at low parasitaemia would be a challenge if only a small fraction of parasites contain detectable haemozoin. A much larger volume of blood must be observed and a correspondingly higher object-level specificity is required to avoid a prohibitive FP rate. Note that because of the window of detection, the keys to successful detection are the parasite age distribution and the location of the distribution in time (i.e. when the blood is drawn from the patient). Similarly, it is possible that a dedicated DF gametocyte detector algorithm might find gametocytes in all synchronized early ring samples. However, because gametocytaemia is typically much lower than parasitaemia, a much higher volume of observed blood and a correspondingly prohibitive FP rate would be required. Only one of the five early ring-synchronized samples in the sample set contained gametocytes.

These results highlight the challenges of haemozoin detection in the peripheral blood for the diagnosis of *P. falciparum*, especially at the very low parasitaemia levels relevant to screening and surveillance use-cases. A haemozoin-based system could potentially serve in other use-cases: for diagnosing non-*P. falciparum* strains (as noted in [1]); in situations where blood can be imaged multiple times at several-hour spacing; or for detecting gametocytes. To establish whether the DF system described here would work in these cases, many more field samples would need to be imaged in order to get patient-level statistics, and in each sample more DF fields of view would need to be imaged in order to assess the parasitaemia detection limit. However, the evident lack of haemozoin under DF in early (*<*6 hours) rings and the consequent problems with *P. falciparum* diagnosis make the authors pessimistic about the diagnostic value of haemozoin-based methods at this time as a tool for malaria case management.

## Competing interests

The authors declare that they have no competing interests.

## Author’s contributions

MCH conceived of the experiment. MCH, MPH, BW, CD, JP participated in the experimental design. MCH and MPH collected the data. CD wrote and performed the image processing. CD, MCH, MPH, BW, JP performed data analysis. CD drafted the manuscript. MCH and MPH critically revised the manuscript. All authors read and approved the final manuscript.

## Supplementary Material

Additional file 1 This PDF contains a detailed description of the image processing and classification algorithms. Click here for file
