# Supplementary material for: Limitations of haemozoin-based diagnosis of Plasmodium falciparum using dark-field microscopy
Source: Malar J. 2014 Apr 17;13:147. doi: 10.1186/1475-2875-13-147 (PMC4021049; doi:10.1186/1475-2875-13-147)
Supplement: Additional file 1 — This PDF contains a detailed description of the image processing and classification algorithms. [file 1475-2875-13-147-S1.pdf]

# Additional File 1: Algorithm Description

## 1 Overview

The hemozoin detection algorithm is based on a) detection of radial symmetry, and b) clustering in HSV color space. This document gives details of the algorithm. Sub-sections include Masking, Radial Symmetry, Color, and Classifier Structure. Figure 1 shows a schematic of the algorithm.

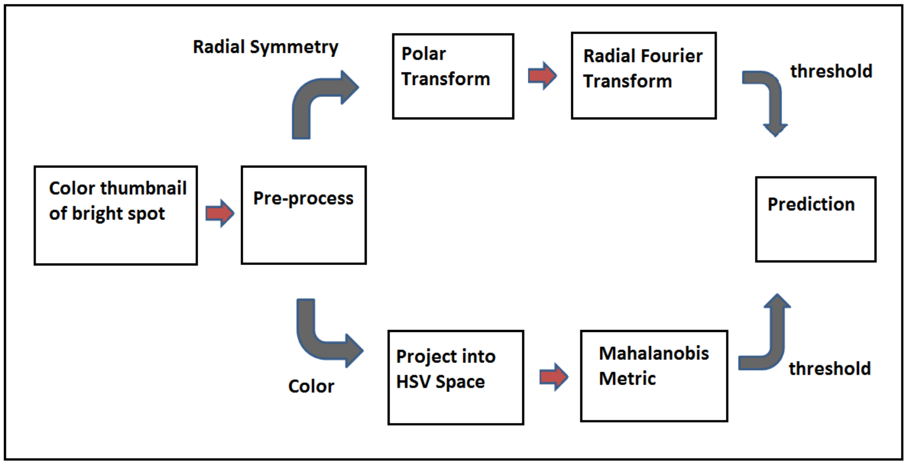


Figure 1: Schematic of diagnosis system. The pre-processed thumbnail is analysed on two parallel tracks, radial symmetry and color. The two results combine to give a classification.

## 2 Masking

Each object is masked by applying an adaptive threshold to its grayscale DF 50x thumbnail *im*. This threshold includes a component proportional to the difference between each image's background level *back(im)* (e.g. the median pixel value of the "empty", or darkest, sections) and a mid-range value $midVal$ (fixed for all images).

$$maskThresh = \ (1-\alpha)back(im) + (\alpha)midVal, \tag{1}$$

where $\alpha$ is a fixed scalar in [0,1].

The goal of *maskThresh* is to capture the relevant signal given the variable exposure levels. Because $midVal$ is fixed (independent of sample), *mask-Thresh* has an upper bound that prevents samples with very high contrast (e.g. very bright spots) having such high thresholds that valuable but fainter information is lost. Also, $midVal$ is high enough to (ideally) ensure that cell information due to hemoglobin is rejected, regardless of the exposure level of the sample.

To mask an image, all pixels with grayscale value > *maskThresh* are retained.The particular method of grayscaling is not critical. The resulting masked image is then centered about the weighted centroid of the largest connected piece. No rotation is applied.

## 3 Shape/Radial Symmetry

Hemozoin forms in crystals spread around a ring-shaped parasite. Thus the masked images of hemozoin typically have irregular shape. In contrast, the images of bubbles and dust particles tend to be radially symmetric. This is the physical basis for using radial symmetry as a feature for classification. There are various methods available to isolate this feature, including use of angular variances, convexity statistics such as $perimeter/area$, and angular Fourier transforms. The angular Fourier transform is discussed here. There are three main steps: Polar Transform; Fourier Transform; and Combining Terms.

#### 3.0.1 Polar Transform

As a first step, each masked, grayscaled object is transformed to polar (angle, radius) coordinates. The polar image has angle (0 to 359.5) along the x-axis,

and radius along the y-axis. See Figure 2.

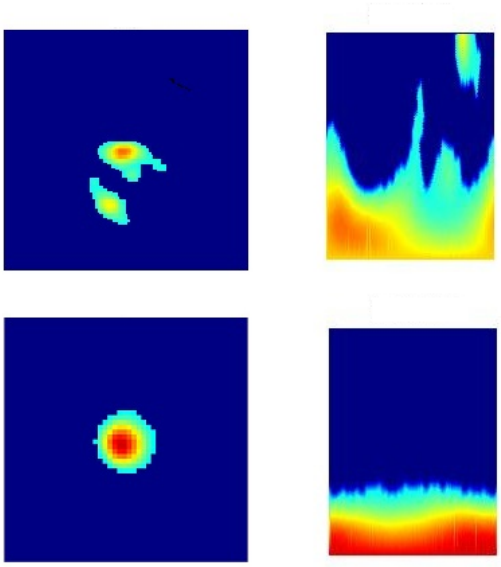

Figure 2: Masked Hemozoin (top) and Distractor (bottom) object images (left), and their polar transforms (right). 0 degrees (the left hand column of the polar image) corresponds to the ray pointing straight left from the center of the Cartesian image.

This transform includes the following steps:
1. 16x upsampling via bilinear interpolation (e.g. Matlab's interp2(image,2));
2. Standard discrete polar transform, in which each pixel x,y maps as

$$(x, y) \Rightarrow (\text{round}(\arctan(y/x)), \text{round}(\sqrt{x^2 + y^2}); \quad (2)$$

3. 25x downsampling via non-zero median of 5x5 blocks, i.e. each 5x5 block maps to the median of its non-zero elements;
4. Bilinear interpolation of each column.

The final vertical interpolation (step 4) is necessary because a discrete (i.e. pixelated) image does not map to the entire polar image space (e.g. there are only 4 pixels at distance 1 from the center, but there are 360 pixels corresponding to radius 1 in the polar image). Vertical interpolation (along the radius axis) is used since the Fourier transform is taken horizontally (along the angle axis).

The radial coordinate of a transformed pixel requires rounding, since the radial distance to a pixel is rarely an integer. Due to interpolations and rounding, the polar transform inevitably introduces noise. The purpose of the up- and downsampling (steps 1 and 3) is to mitigate this noise and smooth the resulting polar transform.

## 3.1 Radial Fourier Transform

The continuous Fourier transform for a function *f(x)* is defined as:

$$F(\theta) = \int f(x)e^{-i\theta x}dx, \quad \theta \in [0, 2\pi) \tag{3}$$

The discrete Fourier transform (including the FFT) at $k \in \mathbb{Z}$ of a vector *f(n)* of length $N$ is defined as:

$$F(k) = \sum_{n=0}^{N-1} f(n)e^{-2\pi ikn/N}, \quad k \in [0, N-1] \tag{4}$$

Given a polar image of an object, a standard FFT is applied along the angle axis (i.e. along each row). This gives an image where each row consists of the Fourier coefficients w.r.t. angle at a fixed radius, i.e. the angular frequency content of the circle with that radius in the image. Since the specific goal here is to detect radial symmetry (i.e. change w.r.t. angle), no Fourier transform is taken along the radius axis (vertically).

In the continuous case, perfect radial symmetry gives pure DC Fourier coefficients. In the discrete case, imperfect radial symmetry of discrete images and noise intrinsic to the polar transform of a discrete image combine to give small, roughly uniform, frequency content at all frequencies for even a "perfect" discrete image circle. However, higher values at low frequencies distinguishes less radially symmetric images.

## 3.2 Combining Terms

To condense the data, coefficients at each frequency are first summed over all radii, giving a 1D vector *fftCoeffs* where each entry corresponds to an angular frequency. Then the cumulative sum,

$$cumulSum(n) = \frac{\sum_{i=1}^{n} \textit{fftCoeffs(i)}}{\sum_{j=0}^{end} \textit{fftCoeffs(j)}}, \tag{5}$$

gives a measure of the proportion of frequency content at or below the $n$'th frequency. The DC component is included in the denominator, but not in the numerator. In the continuous case, perfect radial symmetry would thus give *cumulSum* $\equiv 0$ (since angular frequency content is pure DC). In the discrete case, a flatter curve and the small values of cumulSum correspond to radially symmetric images. See Figure 3.

Because it emphasizes DC and low frequencies, this method gives better discrimination of radial symmetry than simply using the variances of the pixels at each radius, which is equivalent (by Parseval's theorem) to using the full squared sums of the Fourier coefficients of the mean-subtracted pixels at each radius. Also, it allows use of interior pixel information, unlike the statistic $perimeter/area$, which assumes a binary image.

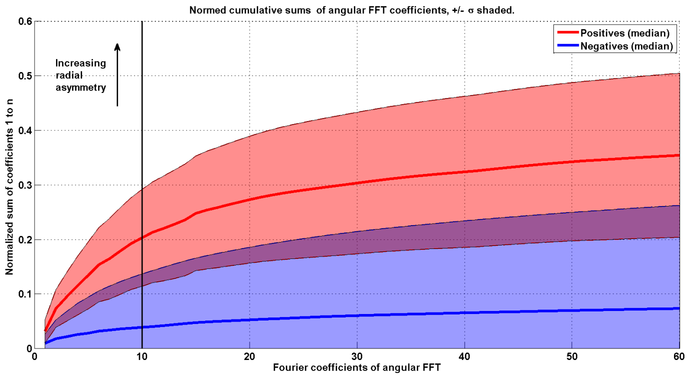


Figure 3: Cumulative sums from the radial FFT. The red band shows the median +/- $\sigma$ for the cumulative sums of hemozoin objects. The blue band shows the median +/- $\sigma$ for the cumulative sums of non-hemozoin objects. Objects with near-perfect radial symmetry have low, flat curves. Objects are thresholded by taking a vertical slice through the graph at $n = 10$. *FTthresh* is applied to these values.

Objects are classified using a simple threshold on the objects' *cumulSums* at some fixed frequency, say $n = 10$. Objects with *cumulSum(n)* $<$ *FTthresh* are classed as a distractor. For example, in Figure 3, a threshold $= 0.1$ with $n = 10$ would cull the bottom tranche (i.e. over half) of negatives as well as 3 positives. *FTthresh* $= 0.16$ would cull the vast majority of negatives as well as 6 positives. *FTthresh* is determined by requiring some minimum sensitivity over a training set (discussed in "Tuning The Classifier" below).

# 4 Color

The crystal hemozoin has particular light-scattering properties. Roughly speaking, hemozoin scatters blue light [3], while objects such as bubbles and dust scatter more uniformly white light. This is the basis for using color as a feature.

To create color-based features, the masked full-color RGB dark field thumbnail is converted to Hue-Saturation-Intensity (Value) coordinates. Note that here "saturation" means "purity of color", not saturation of pixels due to too much light. For clarity we refer to Hue-Purity-Intensity. "Hue" refers to which color; "Intensity" is similar to grayscale value. All values are on [0, 1]. On the Hue axis, 0 = red, 0.3-0.4 = green, and 0.6-0.7 = blue. A pure grayscale pixel (i.e. one with equal values for R, G and B) has Purity = 0 and Hue = 0.

For each object, two statistics are found using the masked pixels: the mean of Hue values (*meanHue*) and the mean of Purity values (*meanPurity*). These statistics use only the non-saturated masked pixels (i.e. pixel's RGB $\neq$ [1,1,1]), which implies Hue and Purity $\neq 0$.

To classify objects using color, *meanHue* and *meanPurity* are first scatter-plotted against each other. Second, the hemozoin objects are clustered using, for example, Expectation-Maximization (E-M, [1]) in the event of two clusters; or E-M (trivially) or principal component analysis (PCA) in the case of one cluster. This gives, for each cluster, a center and a Mahalanobis metric to measure distance from this center (where 1 mahalanobis unit = 1 standard deviation in the Euclidean metric [2]).

Classification using the color scatter-plot is by simple threshold: Let *MahDist* be the Mahalanobis distance of the object's color coordinates from the centroid of the closest hemozoin cluster. Objects with $MahDist > MDthresh$ are classed as distractors.

## 4.1 Types of Hemozoin Objects in Color Space

This section discusses why objects from the saturated (over-exposed) slide were removed from the dataset. Basically, saturating one or more colors in a pixel changes the pixel's clustering in color space.

The original dataset contains 72 hemozoin objects and 946 distractor objects. The hemozoin objects correspond to 6 gametocytes; 1 early ring (0-6 hours); and 64 mid-late rings (> 6 hours), trophozoites and schizonts. 18

of the mid-late ring hemozoin objects come from one sample with an especially high exposure level. As a result, these 18 hemozoin objects images are saturated, i.e. many pixels have values = (1,1,1). The rest of the hemozoin objects are not saturated.

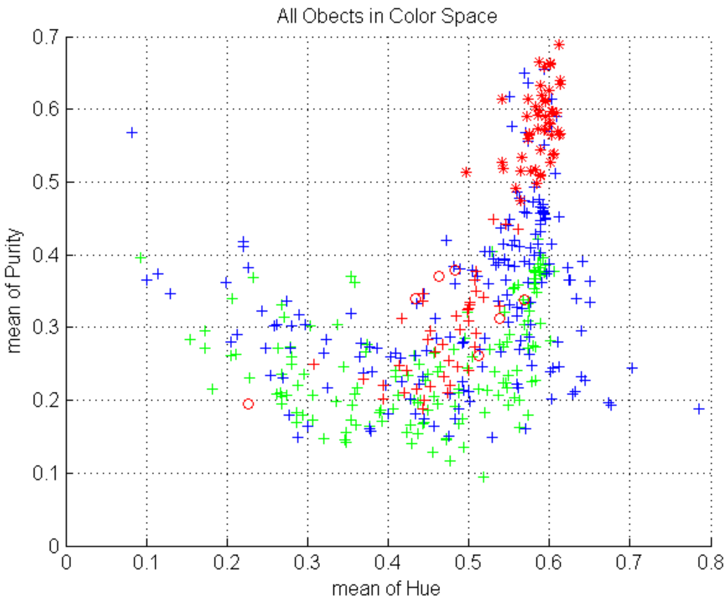


Figure 4: All Objects in Color Space: $x$-axis = Hue of object, $y$-axis = color Purity of object. Blue = distractors (positive samples), Green = distractors (negative samples), Red (+) = hemozoin from over-exposed images, Red (*) = hemozoin from non-saturated images, Red (o) = gametocytes. Note that there are two natural clusters of hemozoin objects. The lower left cluster consists of saturated (over-exposed) hemozoin objects and gametocytes.

When all these objects are mapped into Color space, the hemozoin objects form two distinct clusters (see Figure 4). The top right cluster consists of objects with blue color and high purity.These are precisely the non-saturated (and non-gametocyte) hemozoin objects, i.e. objects from samples where the exposure levels were low enough that the pixels of the objects did not start saturating (RGB values did not reach 1). These objects cluster tightly and are readily separated from distractor objects in Color space. The lower left cluster consists of precisely two types of hemozoin objects: saturated objects from the one slide that had very high exposure level, and gametocytes.

First consider the saturated objects: When an RGB pixel saturates its blue (B) value, its hue and purity decrease (assuming R $<$ G). For example, a pixel with RGB values = (0.28, 0.448, 0.7) is strongly blue (Hue = 0.6, Purity = 0.6). As the pixel saturates, the RGB values become (0.7, 1, 1) since pixel value cannot exceed 1. This is equivalent to Hue = 0.5, Purity = 0.3. Thus, saturating the pixels of an object in the top right cluster moves it down and to the left, into the second cluster. If the imaging protocol is adjusted to avoid over-exposure and saturated pixels, most of the objects in the lower left hemozoin cluster disappear. Saturation of distractor objects does not cause the opposite problem, because distractor object Hue and Purity values are also shifted down and left by pixel saturation.

Next consider the gametocytes. These contain a great deal of hemozoin. They also display a large range of color, perhaps because the crystals are in a wide range of orientations, and hemozoin crystals viewed end-on scatter other colors than blue [3]. Gametocytes are very distinctive, and can be readily detected in various ways. However, the *meanHue* statistic used by the algorithm fails, since it assumes roughly uniform color.

We assume that gametocytes can be identified with a dedicated algorithm (for example, one can combine the first detail of a wavelet analysis of pixel hue with the histograms of pixel hue in an object). Similarly, we assume that saturated hemozoin objects can be avoided with modified device protocols (i.e. limiting exposure times). We therefore remove both these categories from the dataset. The resulting dataset consists of 51 non-saturated hemozoin objects from mid-late rings, trophozoites and schizonts, and 923 distractor objects. For this dataset, the hemozoin objects form one distinct cluster in Color space (see Figure 5, left hand plot) because there are no saturated objects or gametocytes.

# 5 Classifier Structure

The classifier has two stages. In the first, the radial FFT threshold is applied to the test object. If the object is not rejected as a distractor, then in the second stage the Color threshold is applied.

$$(\text{FFTstage} == \text{Distractor}) \; || \; (\text{ColorStage} == \text{Distractor}) \Rightarrow \\ \text{class} = \text{Distractor}. \tag{6}$$

The effect of the AND structure is that objects in the top right of Figure 5 (right hand plot) are labeled as ‘positive’.

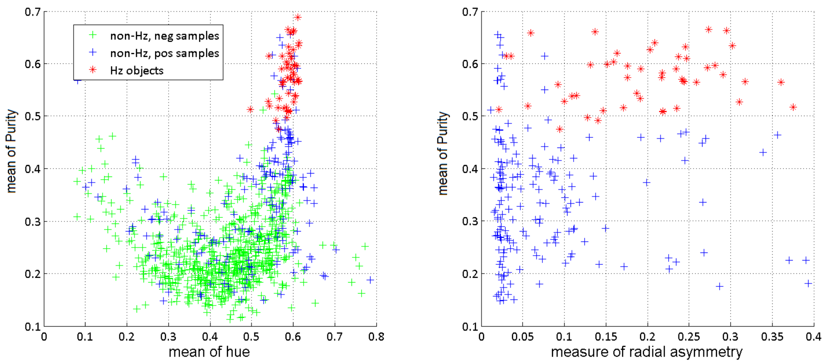


Figure 5: Left hand plot: Color Space plot of modified dataset, i.e. no saturated hemozoin or gametocytes. $x$-axis = Hue, $y$-axis = color Purity. Blue = distractors (positive samples). Green = distractors (negative samples). Red = hemozoin. Note the clear hemozoin cluster. Right hand plot: color Purity vs radial symmetry. $x$-axis = measure of radial symmetry, high being radial asymmetric. $y$-axis = color Purity, identical to the $y$-axis of left hand plot. Red = hemozoin, blue = distractors (positive objects). The few distractor objects with high color Purity have low radial asymmetry. AND structure on the two features thus gives high classification accuracy.

## 5.1 Setting Thresholds

The two thresholds are set by specifying two desired sensitivities, *Desired FFT Sensitivity*, and *Desired Overall Sensitivity*, then applying the classifier to the training set of objects.

First, the FFT threshold is set to be as high as possible while still ensuring that the sensitivity of the FFT stage, when applied to the training set, is greater than the *Desired FFT Sensitivity*:

$$
\begin{aligned}
FTthresh = \max\{&\text{all thresholds that give sensitivity} \geq \\
&\textit{Desired FFT Sensitivity} \text{ on training set}\}
\end{aligned}
\tag{7}
$$

This ensures that the FFT specificity is as high as possible given the tuning constraint *Desired FFT Sensitivity.*

Second, the Color threshold is determined using those training objects that were classed as positive by the FFT stage. The Color threshold is set as low as possible while still ensuring that the algorithm's overall sensitivity $\geq$ *Desired Overall Sensitivity*:

$$
\begin{aligned}
colorThresh = \min\ \{&\text{all thresholds that give sensitivity} \geq \\
&\textit{Desired Overall Sensitivity} \text{ on training set}\}
\end{aligned}
\tag{8}
$$

This ensures that the overall specificity is as high as possible given the tuning constraint *Desired Overall Sensitivity.* In general, high tuning constraints give a classifier with high sensitivity and lower specificity. Low tuning constraints give high specificity and lower sensitivity.

# References

[1] Elements of Statistical Learning 2nd ed, Hastie T, Tibshirani R, Friedman J, Springer, 2009

[2] Encyclopedia of Mathematics, Hazewinkel M, Springer, 2001

[3] Wilson B, Behrend M, Horning M, Hegg M, Detection of malarial byproduct hemozoin utilizing its unique scattering properties, Optics Express 2011, 19(13):12190-12196.

[4] Digital Image Processing 3rd ed, Gonzalez C and Woods W, Prentice Hall, 2007
